# Complexity in action: Untangling latent relationships between land quality, economic structures and socio-spatial patterns in Italy

**DOI:** 10.1371/journal.pone.0177853

**Published:** 2017-06-02

**Authors:** Luca Salvati, Ilaria Tombolini, Roberta Gemmiti, Margherita Carlucci, Sofia Bajocco, Luigi Perini, Agostino Ferrara, Andrea Colantoni

**Affiliations:** 1 Italian Council for Agricultural Research and Economics (CREA), Rome, Italy; 2 Department of Methods and Models for Territory, Economy and Finance "Sapienza" University of Rome, Rome, Italy; 3 Department of Social and Economic Sciences, “Sapienza”, University of Rome, Rome, Italy; 4 School of Agricultural, Forest, Food and Environmental Sciences, University of Basilicata, Potenza, Italy; 5 Tuscia University–(DAFNE) Department of Agricultural and Forestry scieNcEs- Via San Camillo de Lellis, Viterbo, Italy; Public Library of Science, FRANCE

## Abstract

Land quality, a key economic capital supporting local development, is affected by biophysical and anthropogenic factors. Taken as a relevant attribute of economic systems, land quality has shaped the territorial organization of any given region influencing localization of agriculture, industry and settlements. In regions with long-established human-landscape interactions, such as the Mediterranean basin, land quality has determined social disparities and polarization in the use of land, reflecting the action of geographical gradients based on elevation and population density. The present study investigates latent relationships within a large set of indicators profiling local communities and land quality on a fine-grained resolution scale in Italy with the aim to assess the potential impact of land quality on the regional socioeconomic structure. The importance of land quality gradients in the socioeconomic configuration of urban and rural regions was verified analyzing the distribution of 149 socioeconomic and environmental indicators organized in 5 themes and 17 research dimensions. Agriculture, income, education and labour market variables discriminate areas with high land quality from areas with low land quality. While differential land quality in peri-urban areas may reflect conflicts between competing actors, moderate (or low) quality of land in rural districts is associated with depopulation, land abandonment, subsidence agriculture, unemployment and low educational levels. We conclude that the socioeconomic profile of local communities has been influenced by land quality in a different way along urban-rural gradients. Policies integrating environmental and socioeconomic measures are required to consider land quality as a pivotal target for sustainable development. Regional planning will benefit from an in-depth understanding of place-specific relationships between local communities and the environment.

## Introduction

Land is a pivotal capital supporting local development [1]. Land properties depend on the multifaceted interactions between natural factors and human-driven processes [2]. Extensive land-use transformations were observed in the last century with vast impact on land quality [3–8]. These processes include compact and dispersed urbanization, industrialization, infrastructure development and agricultural intensification, as well as economic marginalization and the abandonment of remote rural areas [9–11].

In the light of the increasing concern about soil resources in Europe, and especially in Mediterranean Europe [12], attempts have been made over the last decades to classify land according to increasing levels of quality for agricultural purposes or other productive use (e.g. forestry, pasture or urban use) [13]. In the case of land quality, two approaches have been followed: (i) the assessment of land capability (i.e. the broad potential of the land for cropping, natural vegetation or human settlements) or (ii) the assessment of land suitability, with the objective of defining the maximum productivity of the land for specific crop or other economic uses [14]. Exercises based on both approaches can be found for Spain [4], Italy [15–16] and Greece [16].

Land quality is considered a relevant attribute of local economic systems. It has been hypothesized that natural capital, and especially land quality, have influenced the organization of regional economic systems determining specific location patterns for agricultural, industrial or service activities and, more generally, urban settlements [12–17–18]. In regions with a long-established human-landscape interaction, such as the Mediterranean basin, soil capital and land quality have been also hypothesized to represent key factors at the base of socioeconomic disparities and polarizations in the use of land along both urban-rural and elevation gradients [19–21]. Territorial disparities, economic backwardness, rural poverty and increased pressures on fragile areas have been demonstrated to be decisive in processes of depletion or degradation of land resources [22–25].

Partial and often fragmented evidences were provided to outline the intimate relationship between land capital endowments and specific socioeconomic profiles at either regional or local scale.

Land quality and local development have been marginally explored jointly using holistic approaches [25–27]. Specific exercises were devoted to identify socio-demographic, cultural and institutional variables possibly influencing the level of land vulnerability to degradation over time and space [28]. Imeson [29] reviewed the role of selected (biophysical) drivers of soil degradation in southern Europe. Salvati and Carlucci [30] have proposed a monetary assessment of land quality depletion implementing a procedure based on the user cost approach. Finally, some proximate causes and indirect drivers of land resource depletion have been identified and discussed by Salvati and Zitti [31]. However, these exercises have produced a partial and heterogeneous framework on dynamic links between socioeconomic local structures and land capital endowments [30]. A comprehensive overview of the connections between the intimate characteristics of local communities and the geographical distribution of high-quality land is required in sustainable planning and management of urban and rural regions [32], promoting economic specialization and local competitiveness in a framework of spatial equity and cohesion [33].

Although field surveys have often produced assessments of land quality at the regional or national scale, their ability to meet more general environmental policy needs is rather limited, being neither comprehensive in geographical coverage nor methodologically homogeneous [34]. Most have also been designed for specific land management systems [35], so cannot easily be extrapolated to new areas or applications.

Approaches that go behind the traditional urban-rural divide and focus on the central role of local socioeconomic contexts are especially required in the identification of dynamic patterns underlying complex socio-ecological systems [26,36,37]. In Mediterranean Europe, the socio-spatial structure of local communities is the result of a complex interplay of demographic, cultural, political and economic factors reflecting the millenary interaction between nature and humans [38]. We hypothesize that an in-depth knowledge of territorial characteristics and local community profiles allows assessing latent socioeconomic patterns possibly shaped by the geographical distribution of high- and low-quality land [39]. A comparative analysis of socioeconomic profiles of local communities developed on diverging land quality conditions (high, intermediate, low) provides the required information base for identification of 'sustainability' conditions in local development [23,25,33]. Local contexts with balanced socioeconomic and environmental conditions are meaningful examples for such applications.

The present study assesses the influence of three composite indexes of land quality on different socioeconomic and territorial contexts in Italy. The peculiar urban hierarchy—based on polycentric metropolitan nodes compared to countries with dominant cities, such as France, United Kingdom or even Portugal and Greece—and the geographically-differentiated rural districts make Italy an interesting case for investigating complex socio-environmental systems which reflect the millenary interaction between natural landscapes, cropping systems and local communities [17,21,40]. We considered local communities and the associated socioeconomic context as a multidimensional analysis domain affecting (and in turn being influenced by) land quality [25], taking account of the regional configuration of cropland, human settlements and industrial activities. Based on these premises, we implemented a data mining approach with the aim to identify socioeconomic profiles discriminating the Italian local communities according to the level of land quality. The approach was centred on a multivariate analysis of 149 background indicators at the municipal scale. The analysis of socioeconomic indicators at a very disaggregated geographical scale (e.g. municipalities, homogeneous agricultural regions, local economic districts) allows profiling local communities from the point of view of complex, socio-ecological systems [38].

## Materials and methods

### Study area

Italy extends 301,330 km^2^ with 23% flat areas, 42% uplands and 35% mountainous land [30]. The country is characterized by socioeconomic divides between northern and southern regions, urban and rural areas, coastal and inland districts. Socioeconomic divides reflect spatially-heterogeneous landscape and vegetation types, soils, agricultural systems and climatic regimes, even within relatively small areas [40]. Italian land is administered by twenty regions and more than 8,000 municipalities. The reference spatial unit of this study (8,100 local municipalities) refers to the 2001 administrative asset. This choice allows for the effective matching with several indicators derived from official statistics at the local scale [41]. The local governance system was changed moderately in 2013 maintaining 8,094 municipalities that administer the Italian territory.

### The elementary analysis domain

The use of municipalities and local districts as the elementary spatial domain in the analysis of land quality and socioeconomic profile of rural communities is proposed and discussed by Salvati and Zitti [32] and Serra et al. [42]. Despite some criticisms concerning the use of administrative boundaries for assessing environmental variables [21], these spatial units show appreciable features that fill the need for data integration in ecological, social and economic fields [38]. Indeed, the use of municipal data presents the following advantages: (i) to address the local dimension of social, demographic and economic processes, (ii) to exploit quantitative information available at an enough detailed spatial scale when analysing the impact of land quality on socio-spatial and production structures and (iii) to allow identifying the most relevant geographical gradients supposed to affect configuration of both urban and rural regions [43–45].

### Assessing land quality

The multifaceted relationship between land quality and the socioeconomic local context is difficult to assess objectively because of soil variability, topography, multiple sources of ecological heterogeneity and land-use variety and diversification of socioeconomic activities and functions [15,46,47]. Indeed, land quality can be defined in different ways according to the disciplinary perspective and the final objective of the study e.g. [21,43,48]. Land quality primarily reflects the ability of a soil to sustain agricultural production and/or natural ecosystems [31], considering eventual soil degradation processes at the local scale [21]. On the one hand, land evaluation refers to the specific suitability of land for a given crop, and depends upon a detailed understanding of the environmental (including—but not restricted to—soil) requirements for any specified activity or function [14]. On the other hand, land evaluation may also refer to the much broader capability of the land to support a given (non-agricultural) use, such as urban areas, recreation, pastures or forestry, as well as broader ecosystem services [40]. Limited data availability, the conflicting demand for reliability and generality, and the diversity of conditions at the regional scale have created conceptual and practical difficulties in the development of information systems assessing land quality at the national and continental scale in Europe [35]. Variables derived from official statistical sources, providing homogeneous information over time and space and easily available data at a disaggregated resolution scale (e.g. municipalities, local labour districts, homogeneous agricultural areas) can be regarded as candidate indicators of land quality [30, 32, 49, 50].

Based on these premises, we considered three distinct indicators in the present study: an indicator (LQ) derived from a simplified Land Quality evaluation procedure, a variable assessing land sustainability for agricultural productions based on a specific attribute assessing the maximum potential Available Water Content (AWC) in a given soil, and a more general Soil Quality Index (SQI) quantifying the overall quality of land resources, taking into account soil degradation processes (e.g. erosion, salinization, compaction, sealing, contamination). The selected indicators provide a more comprehensive description of local-scale land quality patterns in Italy than a single-variable approach [39]. Moreover, due to the national coverage of our study, the use of multiple indicators limits the shortcomings associated with the analysis of single variables [39,48,51]. These indexes were considered in earlier works providing a comprehensive assessment of land quality and estimation of soil capital value in Italy and, more generally, in study areas from the northern Mediterranean basin [32,49,52]. Since land quality has been less frequently assessed at the national or continental scale due to e.g. heterogeneity in basic data sources, the comparative use of three widely-used metrics, homogeneous and referenced at the country level, appears as a suitable approach to control for soil's spatial variability [51].

The LQ index was developed on behalf of the land resource evaluation carried out by European Commission (Corine Land Resources) and refers to the Food and Agricultural Organization (FAO) framework for land evaluation which provides a widely-accepted basis for assessing land quality [53]. This approach defines the broad structure for environmental assessment and identifies the main factors that should be considered distinguishing the evaluation of the physical resource base—the inherent quality of land resources—from that of the wider land management system. The LQ index was derived from the computation of thematic variables including (i) soil attributes based on texture, depth and drainage status; (ii) climate quality (calculated as the Bagnouls-Gaussen aridity index, the length of the vegetative period and the frost risk); (iii) topography (indicated by slope angle) and (iv) infrastructural improvements (the presence of permanent irrigation, drainage or terracing). Calculation of the LQ index took place in two stages. In the first step, the potential land quality was calculated based on physical characteristics of the land (soil attributes, climate quality and topography). This provided a measure of the inherent quality of land resource, irrespective of human efforts to improve it. The index was adjusted considering improvements such as irrigation, terracing or drainage with the aim to estimate the actual land quality. The LQ measure represents the current state of land resources taking account of infrastructural factors. Land quality was originally defined on a three-point scale, ranging from 1 (high quality) to 3 (low quality), with an additional class (0) for areas with no inherent quality (e.g. urban areas) and freely disseminated by European Environment Agency (www.eea.europa.eu) as a vector file scaled 1: 100,000 that covers 4 southern European countries (Portugal, Spain, Italy and Greece) and southern France. Indicators were collected from various statistical data sources from national representative organizations (statistical institutes, environmental protection agencies, Ministries of Environment or Agriculture) with late 1990s as the reference period of map production. In the present study, an overall LQ figure was assigned to each Italian municipality by calculating the average of non-urban land quality scores and rescaling the concluding result to the range 0 (low-quality) to 1 (high quality) using an inverse linear transformation. Calculations were run overlapping the LQ map to a vector file provided by Italian National Institute of Statistics (Istat) which contains the boundaries of each Italian municipality at a 1: 25,000 resolution scale [45].

The maximum soil water capacity (sometimes defined as the maximum potentially-Available Water Content of the soil, AWC) is an indicator of a soil’s ability to retain water and make it sufficiently available for plant use [13]. This concept assumes that water readily available to plants is the difference between water content at field capacity and permanent wilting point, based on the intrinsic characteristics of a given soil. Field capacity is the water remaining in a soil after it has been thoroughly saturated and allowed to drain freely, usually for one-to-two days. Permanent wilting point is the moisture content of a soil at which plants wilt and fail to recover when supplied with sufficient moisture. Water capacity is expressed as a depth (mm) or as a volume fraction or percentage [40]. A raster AWC map was derived by [54] from a ‘map of the water capacity in agricultural soils’) generated in the 1990s by the Italian Ministry of Agriculture and based on nearly 18,000 soil samples from the Italian Soil Database updated by the Italian Centre of Pedological Cartography of the Italian Council of Agricultural Research and Economics (Florence: see www.soilmaps.it). Technical details for integrated analysis of soil attributes in Italy include soil water capacity [54]; the final map was made available at a 1: 250,000 scale [40]. An overall AWC figure (mm) was assigned to each Italian municipality by calculating the average of the observed value in the central point of each pixel within a given municipality [47]. Some municipalities especially along the Alps were excluded from the analysis due to the limited availability of soil data.

The Soil Quality Index (SQI) was proposed by Kosmas et al. [13] as a thematic indicator contributing to a decision support system which quantifies land vulnerability to degradation with special regards to Mediterranean Europe based on the Environmental Sensitive Area Index (ESAI) [30]. The SQI was considered in the present study and adopted as a proxy of land quality for its simplified formulation, flexibility and easiness in calculation, and availability at the continental scale in Europe [45]. The indicator was derived from the information contained in the European Soil Database compiled by the Joint Research Centre, Ispra [55]. This information were integrated with data derived from a geo-database of agricultural soil characteristics of Italy, eco-pedological and geological maps of Italy, a land system map produced by the Italian National Centre of Pedological Cartography and a 20 m Digital Elevation Model [40,49,52]. These datasets can be taken as a standard and homogeneous official soil information referring to the mid- and late-1990s made available at 1: 250,000 scale in Italy [21].

The SQI is a composite index based on four variables: parent material, soil depth, texture and slope angle. A set of vulnerability scores was assigned to each elementary variable [39] based on the result of field surveys and bibliographic evidences collected in earlier research [9,13,16,35]. Scores were based on the estimated degree of correlation with independent soil variables measured in selected sampling areas [40]. The SQI was thus estimated as the geometric mean of the different scores attributed to the 4 elementary variables and ranges from 1 (the lowest vulnerability to soil degradation) to 2 (the highest vulnerability to soil degradation). The index was made available in raster format and disseminated at 1 km^2^ resolution. An average SQI score was finally calculated for each municipality by way of the ‘zonal statistics’ tool provided with ArcGIS software (ESRI Inc., Redwoods, USA) after the overlap between the SQI raster file and the shapefile representing the boundaries of each Italian municipality. The 'zonal statistics' procedure computes a surface-weighted average of the raster values corresponding to the central point of each elementary pixel belonging to any given analysis unit [21].

### Contextual indicators

The data used in the present study (see list in S1 Table) were made available from statistical sources (primarily from the Italian National Statistical Institute, Istat) and usually refer to a time period encompassing the early-2000s. This temporal gap represents the most recent point in time with an enough large availability of socioeconomic indicators on a municipal scale in Italy [41]. Changes in census techniques, the unavailability of some variables in the most recent years, dissemination programs encompassing 2015 for other variables prevented us to collect a comparable dataset referring to the most recent period [49]. At the same time, working with early-2000s data allows a direct match with soil data mainly collected at the end of the 1990s and forming the primary informative base for the land quality indicators considered in our study [40]. In this sense, soil attributes, and especially the physical variables considered in this study, remain relatively constant for long times, allowing for a coherent comparison among datasets collected with a temporal misalignment of few years [30,31,40]. A total of 149 indicators have been calculated from the collected variables for each Italian municipality according to [51] and classified into 5 research themes (Population, Economic specialization, Quality of life, Rural development, Environment) and 17 analysis' dimensions (see list in Table 1). Earlier studies profiling Italian local communities from the socioeconomic point of view informed the choice for elementary indicators, analysis' dimensions and research themes [44,49,52]. The selection of indicators suited to assess the territorial context affecting (and, in turn, being influenced by) land quality was based on general criteria of precision, availability, readability and replicability over time and space [21,40,43]. The wide number of thematic dimensions assessed with the selected indicators allows provide a comprehensive overview of the socioeconomic background of Italian municipalities [21,45,56]. A set of indicators assessing the environmental context at the same geographical scale were also considered [51]: the complete list of them, including technical details, statistical data source and reference year, was provided in S1 Table.

**Table 1:** Research themes and analysis dimensions explored by the indicators used (for the complete list of indicators, see S1 Table).

Research theme	Analysis dimension	Number of indicators
Population	Settlement characteristics	11
	Population dynamics/structure	13
Economic specialization	Job market	14
	Education	6
	Economic structure	17
	Tourism	6
Quality of life	Income	6
	Wealth	7
	Crime	5
Rural development	Land tenure	6
	Agricultural landscape	10
	Forests	9
	Innovation and quality in agriculture	9
	Human capital in agriculture	5
Environment	Water use/management	6
	Natural resources	8
	Soil degradation	11

### Data analysis

The geographical distribution of the three indicators (SQI, LQ, AWC) was studied through maps at the municipal scale. Maps are particularly suitable to illustrate heterogeneous distribution of variables possibly reflecting geographical gradients which are hardly described using descriptive or inferential statistics [57]. A data mining exercise including non-parametric correlations and a Principal Component Analysis was developed with the aim of investigating the multivariate relationship between land capital endowments and the socioeconomic profile of local communities at the municipal scale in Italy [32]. Exploratory data analysis are particularly appropriate when analysing the multivariate distribution of a number of indicators assessing a variety of research themes and dimensions [21,49,52]. More specifically, a non-parametric Spearman rank correlation analysis was run to identify significant pair-wise correlations between contextual variables and land quality indicators. Significance was tested at p < 0.01 after Bonferroni's correction for multiple comparisons. To identify the relationship between land quality and the related social, economic and environmental characteristics of local communities, the percentage of significant pair-wise correlations was calculated for each analysis' dimension. A Principal Component Analysis (PCA) was run on the data matrix composed of 149 contextual indicators with the aim to summarize the most relevant factors profiling the multiple socioeconomic contexts of Italy, with the inclusion of the three land quality indicators. The number of relevant axes (m) was chosen by retaining the components with eigenvalue > 4 since the PCA was run decomposing the Pearson correlation matrix and examining the distribution of component loadings (indicators) and scores (municipalities). The contribution of indicators to each component was considered important when the related loading was > |0.5|. Similar thresholds were set up in earlier studies with the final objective of highlighting the most relevant results from the large set of coefficients derived from the analysis [49]. This strategy allowed us to identify the most relevant variables characterizing the socioeconomic profile of Italian local communities, realizing the highest departure from the 'average' multivariate asset in the data matrix. These variables were more likely associated with the highest loadings to the most relevant components extracted by the PCA [58]. Working with subjective, ad-hoc thresholds is a widely-used strategy in multivariate statistics when the final objective of the analysis is eminently exploratory [52]. A non-parametric Spearman analysis was finally run with the aim to identify significant correlations between principal component scores and land quality indicators, testing for significance at p < 0.01 after Bonferroni's correction for multiple comparisons. Moreover, non-parametric correlations allow inferring geographical gradients associated with both land quality indicators and a specific socioeconomic profile identified in the most relevant components of the PCA [49].

## Results

### Descriptive analysis

**Fig 1:**
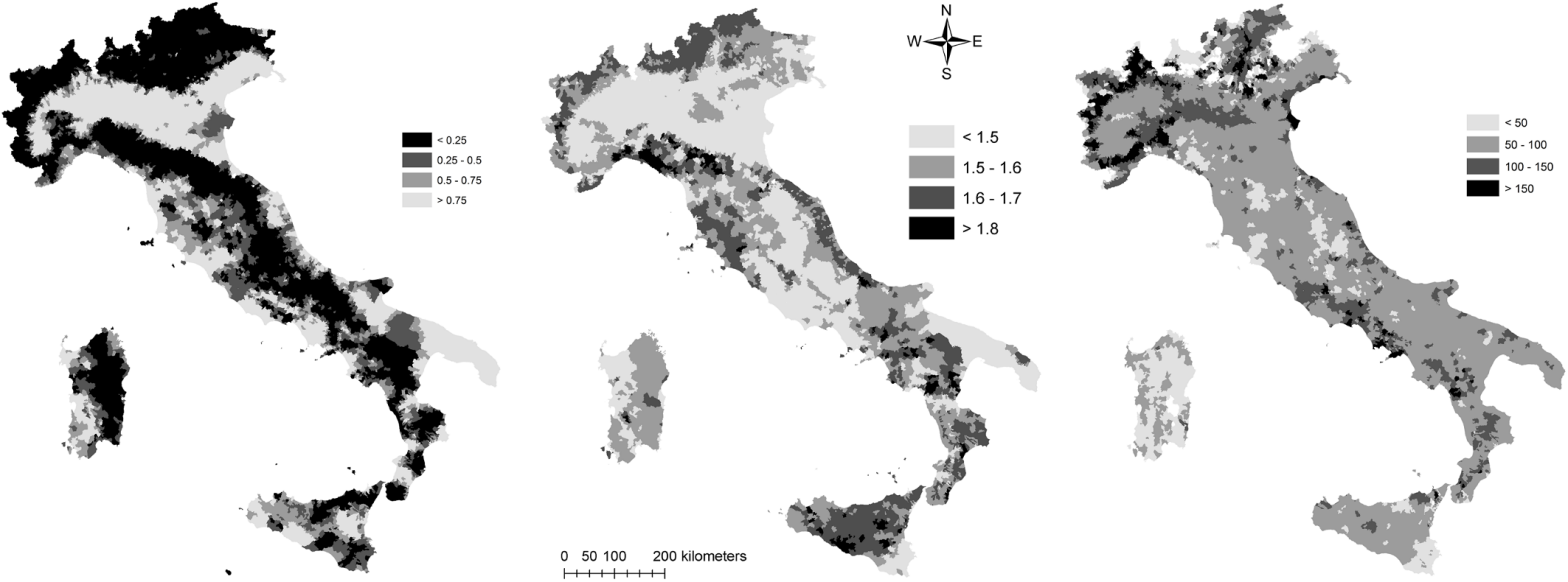
The spatial distribution of three indicators of land quality in Italy (left: land quality index; middle: soil quality index; right: maximum available soil water capacity). Source: own elaboration.

Fig 1 illustrates the distribution of the three indicators of land quality (LQ, AWC, SQI) at the municipal scale in Italy. The LQ index reflects the heterogeneous distribution of high quality land at the country scale, apart from the Po Valley (northern Italy) and Apulia (southern Italy): both areas, devoted to highly-specialized and economically-viable agriculture, display above-average and spatially-homogeneous values of land quality. Soils with high AWC showed a heterogeneous and fragmented distribution; conversely, intermediate AWC values were relatively common for Italian soils. Districts in the western Alps and south-central Tyrrhenian sea coast in Latium and Campania showed systematically higher AWC values than the country average. A large proportion of soils in Sardinia and Friuli-Venezia Giulia was characterized by low or moderately-low AWC values. The geographical distribution of the SQI is quite different from what was observed for LQ and AWC. Areas with low soil quality were concentrated along the Alps, Apennines and the Adriatic Sea coast. Areas with the highest SQI were situated in the Po plain, in central Italy along the Tiber river valley and in Apulia region.

### Correlation analysis

**Fig 2:**
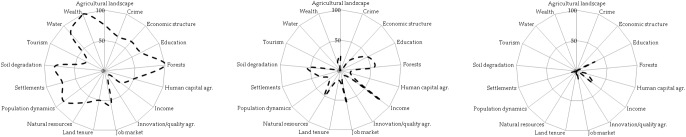
Percentage of significant non-parametric correlations between indicators in each theme and the three independent variables of land quality (left: LQ, middle; AWC, right: SQI). Source: own elaboration.

Based on a Spearman non-parametric rank correlation analysis, the LQ index totalized the highest number of significant correlations with the selected socioeconomic variables (67%), preceding AWC (33%) and SQI (5%). The analysis of pair-wise Spearman coefficients (Fig 2) indicates that the LQ index is associated with variables assessing environmental domains (forests, water, soil degradation, agricultural landscape) and socioeconomic domains (wealth, population dynamics, human settlements). The AWC index correlated significantly with 49 variables assessing 14 out of 17 analysis' dimensions (except crime, tourism and water). The dimensions with the largest number of variables significantly correlated with the LQ index refer to socioeconomic domains (income, job market) and environmental/agricultural research dimensions (forests, soil degradation). The SQI correlated significantly with 8 variables referring to 5 dimensions (education, job market, income, settlements, human capital in agriculture). The analysis of Spearman rank coefficients indicates that the three land quality indicators considered here are not correlated over space. This finding confirms that the indicators selected in our study may provide a comprehensive and non-redundant representation of the distribution of land quality in Italy at a disaggregated geographical scale.

### Multivariate analysis

**Fig 3:**
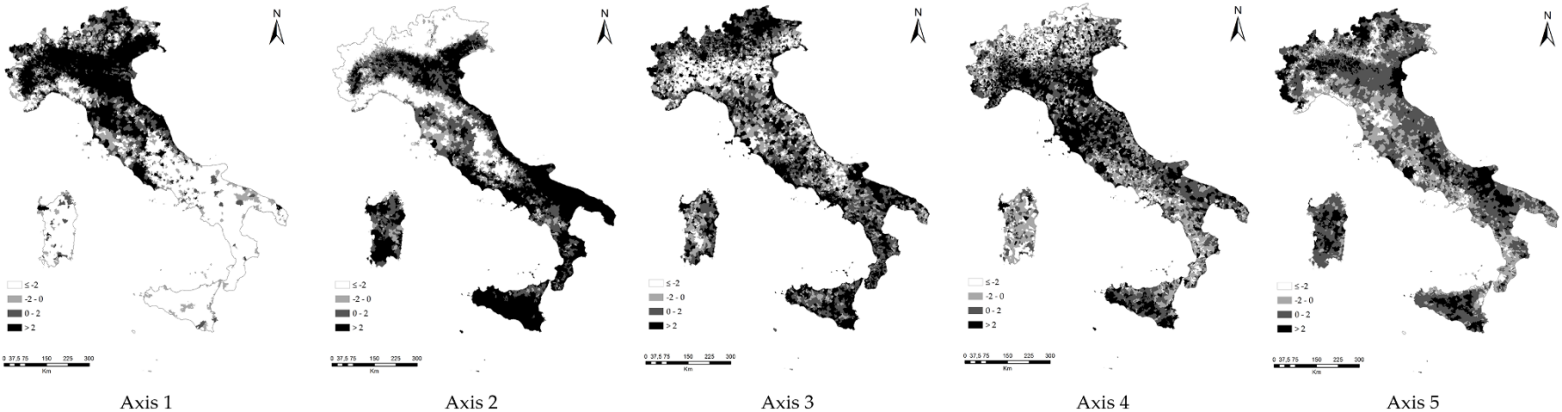
Principal component scores by municipality in Italy. Source: own elaboration.

The PCA run on the entire set of contextual variables extracted 5 components accounting for 34% of the total data variance, considered a relatively high value in respect to the number of input variables (Table 2). The selected components provide a non-redundant representation of the territorial context of Italian local communities. The highest loadings to each component identify the indicators characterizing a specific socioeconomic profile, as illustrated using component scores. Component 1 (12.4%) identifies a typical gradient opposing wealthy to economically-disadvantaged areas in Italy. Wealthy areas were characterized by high population growth rate (1991–2001) and above-average participation and activity rates, number of workers per industrial local unit, percentage of workers in manufacturing, work accidents per 100 inhabitants and sustainable development index. Above-average values of dependency ratio, unemployment rate, unemployment rate of young people, literate population without formal education, illiterate population, percentages of workers in the public sector and in education services characterized economically-depressed districts. Component 1 reflects the traditional north-south socioeconomic gradient in Italy (Fig 3). LQ and AWC indexes correlated positively with Component 1 scores (Table 3). This result indicates the strong association of land quality with demographic and socioeconomic divides in Italy: despite spatial heterogeneity in soil attributes, a high level of land quality was most likely found in wealthy areas and the reverse pattern applies for economically-disadvantaged districts.

**Table 2:** Principal component loadings (see S1 Table for acronyms); loadings > |0.5| are shown.

Variable	Axis 1	Axis 2	Axis 3	Axis 4	Axis 5
I2	0.54				
I5		-0.59			
I6			-0.57		
P1		0.72			
P3				0.56	
P4				0.51	
P5	-0.55				
P9		0.61		-0.53	
L1	0.81				
L2	0.88				
L3	-0.69				
L4	-0.69				
L5	0.80				
L6	0.86				
L7	-0.69	0.51			
L8	-0.68				
F3				-0.58	
F5	-0.61				
F6	-0.64				
S1	0.53				
S6	0.59				
S14	-0.54				
S15	-0.62				
T7		0.52			
Q2	0.65				
Q6	0.51				
Q8	0.83				
Q11	0.69				
Q12	0.51				
Q13	0.55				
D1		0.50			
D4	0.51				
SR-A3					0.52
SR-A5		0.60			
SR-M4		0.73			
SR-Q2					-0.52
SR-Q9					-0.55
SR-P2		0.50			
SR-P3					-0.62
SR-P4		-0.73			
SR-P8		0.60			
Int		-0.64			
Fop		-0.60			
A5		-0.50			
Sdi	0.88				
Ele		-0.61			
Sou	-0.71				
Esa		0.77			
E60		0.50			
Car		-0.80			
Lri		-0.55			
*Expl*. *Var*.*%*	*12*.*4*	*9*.*9*	*5*.*1*	*4*.*0*	*2*.*7*

**Table 3:** Non-parametric Spearman correlation between principal component scores (axis 1–5) and independent land quality variables.

Variable	Axis 1	Axis 2	Axis 3	Axis 4	Axis 5	Sqi	Lq
Sqi	-0.03	0.04	0.03	0.01	0.02	-	-
Lq	**0.41**	**0.58**	-0.15	0.04	**0.20**	0.03	-
Awc	**0.22**	-0.11	0.02	-0.07	**-0.16**	-0.01	0.08

Bold indicates significant correlation at *p* < 0.01 after Bonferroni's correction for multiple comparison

Component 2 (9.9%) identifies the elevation gradient in Italy. Positive component scores were observed in areas situated along the Po river valley in northern Italy, in lowland and mild upland of Apulia and Sicily and along the sea coast in both central and southern Italy, including Sardinia (Fig 3). Negative component scores were associated to mountain districts situated prevalently along the Alps and the Apennines. Flat and hilly areas were characterized by above-average values of number of household components, families with children, crime intensity, agricultural utilized area, crop intensity and percentage of arable land in the agricultural utilized area. Environmental Sensitivity Area Index (ESAI) and its long-term changes over time (1960–1990) were systematically higher in these areas than in the neighbouring districts. A high proportion of agricultural and forest land, non-occupied houses, together with above-average values of forest intensity, number of water reservoirs, topsoil organic carbon content and landslide risk index were typically observed in mountain areas. The geographical distribution of the LQ index was positively correlated with Component 2 scores (Table 3) indicating that land quality is associated with the elevation divide in Italy.

Component 3 (5.1%) is associated to the geographical distribution of the average house size per inhabitant; component scores discriminate compact and semi-dense urban settlements (concentrated along the sea coast in central and southern Italy) from dispersed and discontinuous settlements, mainly situated in the Po river valley. Component scores were not correlated with any indicator of land quality. Component 4 (4%) identified areas experiencing rapid processes of population aging (some parts of Liguria, Emilia Romagna, Tuscany, central and southern Apennines, south-western Sicily). Elderly indexes received positive loadings. The highest negative loadings were attributed to variables such as the percent numbers of families with children and of residents with secondary education. Component 4 scores were not correlated with any of the selected land quality indicators investigated in our study. Component 5 (2.7%) identified a geographical gradient from extensive to intensive cropping systems. The average farm size received a positive loading, in contrast with the percentage of perennial crops, the percentage of land cultivated with local grapevine denominations and the index of economic marginalization of farms, all receiving negative loadings. The LQ index was positively correlated with component 5 scores; the reverse pattern was observed for the AWC. These evidences suggest that a high land quality is an important requisite of intensive cropping systems. Extensive agriculture with high quality productions were associated with fertile soils with high water storage capacity.

Taken together, a high level of land quality at the local scale in Italy is associated with rural communities displaying a characteristic socioeconomic and territorial profile (young population and large families, high household income, intensive agriculture and environmental sensitivity to human pressure). Results of the multivariate analysis also indicate that profiles of municipalities with either low or high land quality in Italy were characterized primarily by three socioeconomic gradients acting at different geographical scales and reflecting (i) the regional differentiation in income, education and job market, (ii) the elevation divide (distinguishing coastal from inland districts) and, finally, (iii) the urban-rural gap.

## Discussion

Land quality, although about other factors, is regarded as a powerful driver of economic growth in both wealthy and emerging regions, shaping socio-spatial structures and long-term demographic dynamics [59]. Although development studies have often evaluated the importance of land as a production factor, there is further scope for exploratory exercises investigating the influence of land quality on the socioeconomic structure of a given region using comparative and spatially-disaggregated approaches [17]. Regional structures are the result of fine interactions between morphological properties and socioeconomic functions in turn depending on a complex interplay of environmental, productive, cultural and political attributes [17].

The exploratory data analysis developed in the present study investigated the multifaceted relationship between socioeconomic factors and territorial conditions underlying land quality in Italy. The joint analysis of three land quality indicators reflecting different definitions and multiple research domains, suggest that LQ is the index correlated with the largest number of socioeconomic attributes profiling local communities. The concept behind the operational definition of the LQ refers to a comprehensive assessment of land quality, considering together the productive, social and environmental services provided by land [52]. The AWC index is correlated primarily with agricultural and territorial variables. These evidences candidate AWC as a typical proxy of land suitability for cropping [49]. The SQI correlated with some territorial variables and this confirms its relevance in the assessment of environmental sustainability [48]. This index characterizes economically-marginal contexts, although being poorly correlated with the distribution of human settlements and the associated socioeconomic dynamics.

Taken together, our analysis identifies elevation and latitude as the geographical gradients most influencing the distribution of land quality in Italy, shaping the socioeconomic profile of local communities at the same time. The distribution of areas with higher land quality is associated with better socioeconomic conditions. Because of the long-term interaction between landscape and local communities, areas with high land quality have allowed the establishment of competitive economic systems, displaying higher per-capita income and lower unemployment rate than the neighbouring districts, albeit with negative environmental externalities [38]. Results from the PCA may confirm this statement, indicating that areas with high levels of land quality are typically characterized by a high level of personal income and a dynamic economic structure.

The relationship between land quality and the socioeconomic context in Italy foreshadows a typical multi-domain problem that invests across the different dimensions of sustainability [60]. On the one hand, environmental conservation policies are required to preserve high-quality land from dispersed urbanization, soil depletion and landscape fragmentation [6,61,62]. On the other hand, an integrated strategy for sustainable development should be implemented with the aim at promoting social cohesion, economic growth and environmental protection in areas with low-quality land [16,24,25]. These areas should be regarded as truly disadvantaged from all points of view of sustainability [1].

It was demonstrated [39] that conditions for a spatially-balanced sustainable development are more likely to be achieved in contexts where land quality (and hence the 'natural' capital) is high. Natural capital availability means a greater chance of developing a balanced socioeconomic context and vice versa. The endowment of natural capital is a key variable in the socioeconomic analysis of local communities in places where the landscape has been shaped (and more or less altered) by long-term interactions between man and the environment [2]. The competitive advantage provided by a higher endowment of natural capital (which may reflect an economically-dynamic local context) should be properly managed with appropriate policies mitigating the negative externalities generated by the local economic system [18]. This justifies a sustainability strategy that designs truly integrated development and environmental mitigation policies which act on the three pillars of sustainability, implementing synergic measures for e.g. agricultural development, deprived districts and protected areas [33].

Taken as relevant measures for eco-compatible land management, integrated policies for sustainable development should promote the adoption of land-use systems that enable land to maximize economic and social benefits, minimize land degradation and rehabilitate degraded areas, while maintaining or enhancing the ecological support functions of land resources [8]. The active participation of local communities in mitigation actions against land degradation is regarded as a crucial point to assure effectiveness of the sustainable development policies. In addition, exploring the intimate structure of complex socio-environmental systems and investigating the relationship between biophysical variables and population, social and economic structure is a requisite for the analysis of future trends in regional development.

## Conclusions

The novelty of this study lies in the use of a vast set of indicators covering homogeneously large areas at a disaggregated geographical scale. Using a data mining approach, the indicators selected have provided a comprehensive profile of the socioeconomic, cultural, political and territorial structure of Italian municipalities and represent a comprehensive information base for geographical systems supporting integrated (socioeconomic and environmental) policy decisions [24,29,59]. Results of our analysis also provide information to design effective responses to preserve priority habitats and high-quality land from e.g. human-driven consumption or degradation processes [33,57,63]. The exploratory multivariate data framework proposed here was considered suitable to investigate the geographical pattern of several contextual indicators. While correlation does not necessarily imply causation, multidimensional and non-parametric statistical techniques contribute to identify non-linear, latent relationships reflecting the complexity of socio-environmental local systems [21].

Evidences collected in the present study outline the complex relationship between socioeconomic profiles of local communities and the environmental features characteristic of Italian land. Our results suggest a ‘turning back’ to the economic geography of land resources when analysing the environment-economy relationship at the local scale [31]. We have demonstrated that a multidimensional approach is particularly suited to grasp the latent interconnection between the different components of socio-environmental systems. Our framework contributes to depict a comprehensive vision of the future path of development towards the attainment of a dynamic equilibrium between the three domains of sustainability. In this view, the multifaceted relationship between factors characterizing local economic structures (e.g. agricultural intensity, industrial concentration, tourism) and the distribution of land quality deserves further investigation. An in-depth understanding of land characteristics from different (e.g. productive, ecological, social) perspectives and a thorough profiling of local communities—intended as complex socio-ecological systems with place-specific relationship with land capital—contribute to develop scenarios' modelling and integrated policies targeting human-driven processes of change. Policies promoting (or improving) sustainability of local development processes require considering imbalances in the spatial distribution of natural capital and differential rates of land consumption (or degradation) caused by the joint action of socioeconomic drivers.

## Supporting information

S1 Table Appendix. The list of indicators used in the present study.
(DOC)
