# Supplementary material for: Complexity in action: Untangling latent relationships between land quality, economic structures and socio-spatial patterns in Italy
Source: PLoS One. 2017 Jun 2;12(6):e0177853. doi: 10.1371/journal.pone.0177853 (PMC5456058; doi:10.1371/journal.pone.0177853)
Supplement: S1 Table — The list of indicators used in the present study. (DOC) [file pone.0177853.s001.doc]

**S1 Table.** Appendix. The list of indicators used in the present study.

| Acronym | Name | Domain | Source | Year |
| --- | --- | --- | --- | --- |
| *Demography and human settlements* | | | | |
| I1 | Population density (inhabitants/km^2^) | Population dynamics/ | Census of population | 2001 |
| I2 | Population growth rate (% per year, 1991-2001) | structure | Census of population | 2001 |
| I3 | Population growth rate (% per year, 2001-2011) |  |  | 2011 |
| I4 | Total municipal footprint (km^-2^) | Settlements | Censuses of population, agriculture, industry | 2001 |
| I5 | Non-occupied houses (%) |  | Census of population | 2001 |
| I6 | Average house size per inhabitant (m^2^) |  |  |  |
| I7 | Compact urban settlements (% on total area) |  | Corine Land Cover map | 2000 |
| I8 | Population residing in urban centers and nuclei (%) |  | Census of population | 2001 |
| I9 | Average municipal surface area (km^2^) |  | Territorial statistics | 2001 |
| I10 | Dwellings built-up later than 1991 (% on total dwellings) |  | Census of buildings | 2001 |
| I11 | Property dwellings (% on total dwellings) |  | | | | |
| I12 | Average surface of dwellings (m^2^) |  | | | | |
| I13 | Head municipality of a Travel-to-work district |  | Territorial statistics | 2001 |
| I14 | Self-commuting (% people working/residing same municipal.) |  | Census of population |  |
| P1 | Number of components per family | Population dynamics/ | Census of population | 2001 |
| P2 | Population > 80 years / births | structure |  |  |
| P3 | Population > 75 years (%) |  |  |  |
| P4 | Elderly index |  |  |  |
| P5 | Dependency ratio |  |  |  |
| P6 | Resident foreign people per 100 inhabitants |  |  |  |
| P7 | Masculinity ratio |  |  |  |
| P8 | Migration component of demographic balance (%) |  | Population register | 2003 |
| P9 | Families with children (% on total families) |  | Census of population | 2001 |
| P10 | Civil weddings (% on total weddings) |  | Population register | 2004 |
| *Labour and education* | | | | |
| L1 | Participation rate | Job market | Census of population | 2001 |
| L2 | Activity rate |  |  |  |
| L3 | Unemployment rate |  |  |  |
| L4 | Unemplyment rate of young people (< 35 years) |  |  |  |
| L5 | Female participation rate |  |  |  |
| L6 | Female activity rate |  |  |  |
| L7 | Female unemployment rate |  |  |  |
| L8 | Unemplyment rate of young women (< 35 years) |  |  |  |
| L9 | Employees on total workers (%) |  | Census of Industry and Services | 2001 |
| L10 | Women workers on total workers (%) |  |  |  |
| L11 | Consultants on total workers (%) |  |  |  |
| L12 | Temporary workers on total workers (%) |  |  |  |
| L13 | Apprentices on total workers (%) |  |  |  |
| L14 | Temporary workers on consultants (%) |  |  |  |
| F1 | Population with tertiary education (%) | Education | Census of population | 2001 |
| F2 | Population graduated in high-school (%) |  |  |  |
| F3 | Population with secundary education (%) |  |  |  |
| F4 | Population with primary education (%) |  |  |  |
| F5 | Literate population without formal education degree (%) |  |  |  |
| F6 | Illiterate population (%) |  |  |  |

Appendix 1. (follows).

| Acronym | Name | Dimension | Source | Year |
| --- | --- | --- | --- | --- |
| *Economy* | | | | |
| S1 | Average number of workers per industrial local unit | Economic structure | Census of Industry and Services | 2001 |
| S2 | Density of workers per municipality surface area (km^2^) |  |  |  |
| S3 | Workers in the agricultural and forestry sectors (%) |  |  |  |
| S4 | Workers in fishing and complementary activities (%) |  |  |  |
| S5 | Workers in the mining sector (%) |  |  |  |
| S6 | Workers in manufacturing (%) |  |  |  |
| S7 | Workers in energy production and distribution industries (%) |  |  |  |
| S8 | Workers in constructions (%) |  |  |  |
| S9 | Workers in the commerce sector (%) |  |  |  |
| S10 | Workers in hotels and restaurants (%) |  |  |  |
| S11 | Workers in transportation and logistics (%) |  |  |  |
| S12 | Workers in financial, insurance and banking services (%) |  |  |  |
| S13 | Workers in informatics, renting and real estate services (%) |  |  |  |
| S14 | Workers in the public sector (%) |  |  |  |
| S15 | Workers in education services (%) |  |  |  |
| S16 | Workers in the health sector (%) |  |  |  |
| T1 | Number of beds in hotels and campings / resident population | Tourism | Census of Industry and Services | 2001 |
| T2 | Average number of beds per hotel |  |  |  |
| T3 | Hotel occupancy level (five-years average) |  | Istat (2006) | 2001 |
| T4 | Camping occupancy level (five-years average) |  |  |  |
| T5 | Agri-tourism occupancy level (five-years average) |  |  |  |
| T6 | Number of beds in agri-tourism accomodation / beds in hotel |  |  |  |
| T7 | Resident population / total number of stores | Economic structure |  | 2000 |
| *Quality of life* | | | | |
| Q1 | Subscriptions on state radio-television channels (%) | Wealth | Banca d'Italia and Istituto Tagliacarne | 1999 |
| Q2 | Number of cars / inhabitants |  |  |  |
| Q3 | Number of deposits / banks |  |  |  |
| Q4 | Number of deposits / inhabitants |  |  |  |
| Q5 | Value of bank deposits / banks (euros) |  |  |  |
| Q6 | Average value of bank deposits (euros) |  |  |  |
| Q7 | Value of bank deposits / inhabitants (euros) |  |  |  |
| Q8 | Per capita income tax amount (euros) | Income | Istituto Tagliacarne | 1998 |
| Q9 | Per capita real estate tax amount (euros) |  |  |  |
| Q10 | Per capita municipal solid waste tax amount (euros) |  |  |  |
| Q11 | Per capita disposable income (euros) |  |  | 2000 |
| Q12 | Per capita consumption (euros) |  |  |  |
| Q13 | Total value added per municipality (euros) |  | CENSIS | 2003 |
| D1 | Crime intensity index | Crime | Istat (2006) | 2000 |
| D2 | Crime severity index |  |  |  |
| D3 | Number of crimes per 1000 inhabitants |  |  |  |
| D4 | Work accidents per 100 inhabitants |  |  |  |
| D5 | Car accidents/total vehicles |  |  | 2002 |

Appendix 1. (follows).

| Acronym | Name | Dimension | Source | Year |
| --- | --- | --- | --- | --- |
| *Agriculture and rural development* | | | | |
| SR-A1 | Rented agricultural surface area / total agricultural surface area (%) | Land tenure | Census of agriculture | 2000 |
| SR-A2 | Agricultural land owned by the state (%) |  |  |  |
| SR-A3 | Average farm size (hectares) |  |  |  |
| SR-A4 | Total agricultural land / Total municipal surface area (%) |  |  |  |
| SR-A5 | Agricultural utilized area / Total agricultural land (%) |  |  |  |
| SR-A6 | Pielou's evenness on farm size distribution |  |  |  |
| SR-Q1 | Agricultural utilized area under organic farming (%) | Innovation and quality and innovation |  | 2000 |
| SR-Q2 | Area cultivated with DOC designation of origin grapevines (%) |  |  |  |
| SR-Q3 | Area cultivated with DOCG designation of origin grapevines (%) |  |  |  |
| SR-Q4 | Livestock organic farms / Total farms (%) |  |  |  |
| SR-Q5 | Agricultural utilized area under good agronomic practices (%) |  |  |  |
| SR-Q6 | Agricultural utilized area under sustainability certification (%) |  |  |  |
| SR-Q7 | Density of cattle (% on total agricultural utilized area) |  |  |  |
| SR-Q8 | Agricultural utilized area applying sustainable irrigation (%) |  |  |  |
| SR-Q9 | Index of economic marginalization of farms |  |  |  |
| SR-Q10 | Density of agro-tourism activities (farms/hectare) |  |  |  |
| SR-L1 | Employees in the primary sector (%) | Human capital |  | 2000 |
| SR-L2 | Farmholders > 55 years (%) |  |  |  |
| SR-L3 | Farmholders on total workers in the primary sectors (%) |  |  |  |
| SR-L4 | Farmholders with technical (agronomy) education (%) |  |  |  |
| SR-L5 | Farmholder's activity diversification index |  |  |  |
| SR-L6 | Family farms (%) |  |  |  |
|  | | | | |
| SR-P1 | Agricultural utilized area under environmental protection (%) | Agricultural landscape |  | 2000 |
| SR-P2 | Arable land / Agricultural utilized area (%) |  |  |  |
| SR-P3 | Perennial crop / Agricultural utilized area (%) |  |  |  |
| SR-P4 | Pastures and meadows / Total agricultural land (%) |  |  |  |
| SR-P5 | Farm size diversity (Shannon index) |  |  |  |
| SR-P6 | Woodland (%on total farm area) |  |  |  |
| SR-P7 | Change in agricultural utilized area (1990-2000, %) |  |  |  |
| SR-P8 | Agricultural landscape diversity (Shannon index) |  |  |  |
| SR-M1 | Number of agricultural machines per farm |  |  | 2000 |
| SR-M2 | Irrigated land / Total agricultural utilized area (%) |  |  |  |
| SR-M3 | Agricultural utilized area per worker in agriculture (ha) |  |  |  |
| SR-M4 | Crop intensity index |  |  |  |
| Int | Forest intensity (%) | Forests | Elaboration on Hansen et al. (2014) map | 2000 |
| Fop | Forest land cover (%) |  |  |  |
| Losst | Forest loss on total land (2000-2012, %) |  |  |  |
| Gaint | Forest gain on total land (2000-2012, %) |  |  |  |
| Lossf | Forest loss on wooded land (2000-2012, %) |  |  |  |
| Gainf | Forest gain on wooded land (2000-2012, %) |  |  |  |
| Fir | Surface area affected by fire (% on the municipal area) |  | Istat (2006) | 2001 |
| For | Burnt woodland (% on total area affected by fire) |  |  |  |
| Exp | Costs for (re)forestation of fire-affected woodland (euros per km^2^) |  |  |  |
| A1 | Per capita distributed water | Water use/management | Census of water resources | 1999 |
| A2 | Water dispersion index |  |  |  |
| A3 | Consumed water/inhabitants |  |  |  |
| A4 | Proportion of water distributed to civil uses |  |  |  |
| A5 | Number of reservoirs (per 100 inhabitants) |  |  |  |
| A6 | Reservoir capacity (per 100 inhabitants) |  |  |  |

Appendix 1. (follows).

| Acronym | Name | Dimension | Source | Year |
| --- | --- | --- | --- | --- |
| *Environment* | | | | |
| Esa | Environmentally Sensitive Area Index (ESAI, 2000) | Nat. resources/territory | Salvati et al. (2014) | 2000 |
| E60 | Long-term change in the ESAI (1960-1990, %) |  |  | 1990 |
| E90 | Short-term change in the ESAI (1990-2000, %) |  |  | 2000 |
| E10 | Short-term change in the ESAI (2000-2010, %) |  |  | 2010 |
| Pro | Protected land (%) |  | Italian Ministry of the Environment | 2000 |
| Sdi | Sustainable development index (score, 0-1) |  | Salvati and Carlucci (2014) | 2001 |
| Ele | Average municipal elevation (m) |  | Istat (2006) | 2000 |
| Sou | Latitude gradient (0: north-centre, 1: south Italy) |  |  |  |
| Car | Topsoil organic carbon content (%) | Soil degradation | European Soil Bureau (JRC) | 2000 |
| Som | Soil organic carbon stock of agricultural soils index |  |  |  |
| pH | Soil ph |  |  |  |
| Con | Potential soil contamination footprint (equiv. inhab/km^2^) |  | Salvati et al. (2011) | 2000 |
| Lro | Landslide risk index |  | European Soil Bureau (JRC) | 2000 |
| Mec | Agricultural pressure to soil compaction index |  |  |  |
| Sus | Natural susceptibility to soil compaction index |  | Salvati et al. (2011) | 2000 |
| Pas | Environmental pressure from grazing index |  | European Soil Bureau (JRC) | 2000 |
| Sal | Soil salinization vulnerability index |  | Salvati et al. (2011) | 2000 |
| Sar | Soil salinization risk index |  | European Soil Bureau (JRC) | 2000 |
| Ero | Soil erosion risk index |  |  |  |
| Sqi | Soil Quality Index |  |  |  |
| Lq | Land Quality index |  | European Environment Agency | 2000 |
| Awc | Maximum potential soil water content (mm) |  | National Centre of Pedological Cartography | 2000 |
